# Antiviral Compounds from Natural Sources Against Human Arboviruses: An Updated Review Including Illustrative In Silico Analysis

**DOI:** 10.3390/pathogens14111156

**Published:** 2025-11-13

**Authors:** Julio Aguiar-Pech, Rocío Borges-Argáez, Henry Puerta-Guardo

**Affiliations:** 1 Unidad de Biotecnología, Centro de Investigación Científica de Yucatán, Calle 43 Número 130 x 32 y 34, Merida 97205, Yucatán, Mexico; rborges@cicy.mx; 2 Virology Laboratory, Centro de Investigaciones Regionales “Dr. Hideyo Noguchi”, Universidad Autónoma de Yucatán (UADY), Merida 97225, Yucatán, Mexico

**Keywords:** arboviruses, DENV, ZIKV, natural compounds, quinones, flavonoids, phenolic, terpenoids, molecular docking

## Abstract

Arboviruses such as dengue (DENV), Zika (ZIKV), and chikungunya (CHIKV) remain major global health threats, especially in tropical regions, with no effective antiviral treatments available. Recent research highlights progress in identifying antiviral compounds from natural sources against arboviruses belonging to the flavivirus genus, such as DENV and ZIKV. These compounds, derived from plants, marine organisms, and microorganisms, fall into several key chemical classes: quinones, flavonoids, phenolics, terpenoids, and alkaloids. Quinones inhibit viral entry and replication by targeting envelope proteins and proteases. Flavonoids disrupt RNA synthesis and show virucidal activity. Phenolic compounds reduce expression of non-structural proteins and inhibit enzyme function. Terpenoids demonstrate broad-spectrum activity against multiple arboviruses, while alkaloids interfere with early infection stages or viral enzymes. To support the reviewed literature, we performed molecular docking analyses of selected natural compounds and some arboviral proteins included as illustrative examples. These analyses support the structure–activity relationships reported for some natural compounds and highlight their potential interactions with essential viral targets such as the NS2B-NS3 protease and NS5 polymerase. Together, these literature and computational insights highlight the potential of natural products as scaffolds for antiviral drug development.

## 1. Introduction

Arthropod-borne viruses (arboviruses) comprise a diverse group of virus families, including *Togaviridae*, *Reoviridae*, *Bunyaviridae*, and *Flaviviridae*, with a transmission cycle involving vertebrate hosts and arthropod vectors such as mosquitoes or ticks [1]. Currently, more than 600 arboviruses have been described worldwide, with more than 30% being responsible for millions of symptomatic infections every year in human populations, particularly in tropical and subtropical regions, where more than 40% of people live [2]. In the case of the mosquito-borne viruses, *Aedes aegypti* and *Aedes albopictus* serve as competent vectors in their transmission cycle, facilitating their spread and persistence, with increasing seasonal outbreaks occurring every year in Africa, Asia, and Latin America [3].

Among the arboviruses, the *Flaviviridae* family is considered the most wanted viral family, encompassing over 70 viral species grouped into four genera: *Hepacivirus* (e.g., hepatitis C virus), *Pestivirus* (e.g., bovine viral diarrhea virus), *Pegivirus* (e.g., GBV-A, GBV-D, GBV-C), and *Orthoflavivirus*. However, other emerging arboviruses that also threaten human public health belong to the *Alphavirus* (e.g., CHIKV) and the *Bunyavirus* (e.g., Oropuche virus, OROV) genera [4]. Overall, these viruses pose substantial public health concerns, underscoring the importance of understanding their molecular characteristics, transmission dynamics, and potential therapeutic targets to develop effective interventions [3].

**Figure 1:**
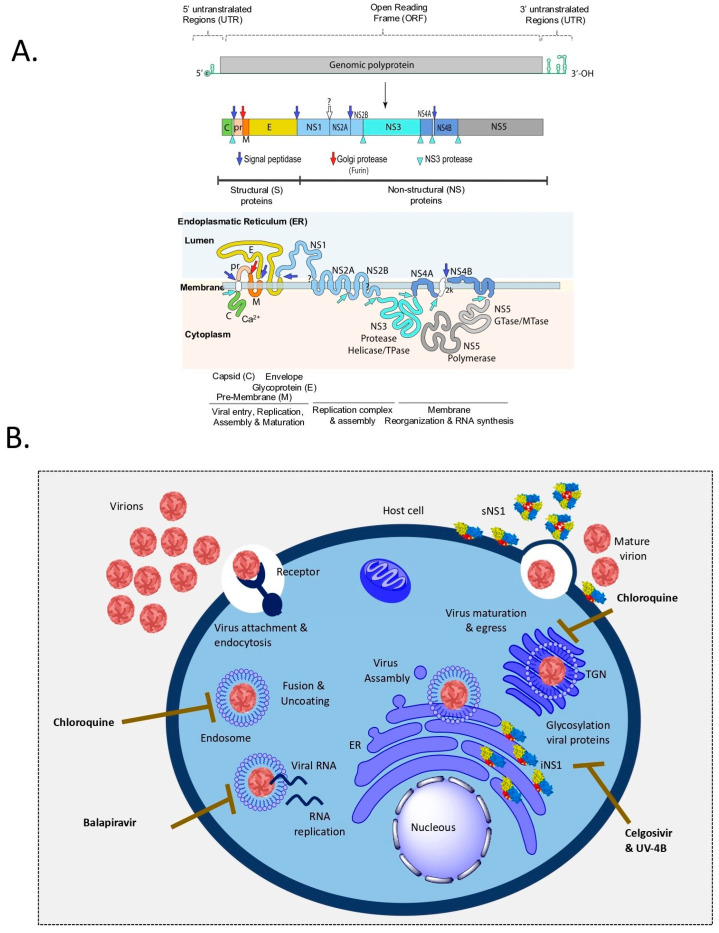
Genome organization, viral proteins, and replication cycle of *Orthoflavivirus*. (**A**) The *Orthoflavivirus* genome is a positive-sense single-stranded RNA (approximately 11 kb) that contains a single open reading frame (ORF) encoding a viral polyprotein, flanked by two 5′ and 3′ untranslated regions (UTRs). The 5′ end has a type 1 cap, but the 3′ end lacks a poly-A tail. The ORF is translated into a polyprotein, which is then cleaved co- and post-translationally into structural (C, prM, E) and non-structural (NS1–NS5) proteins by host and viral proteases as indicated by the arrows in section (**A**). Some proteases are still unknown (?). The UTRs contain conserved structural elements vital for the viral life cycle, including RNA replication and translation initiation. (**B**) Viral infection initiates after the envelope protein of infectious virions interacts with its cognate receptor expressed on the surface of susceptible host cells, leading to virus endocytosis and release of the viral capsid and viral genome (ss + RNA) into the cytoplasm. The translation and cleavage of the viral polyprotein result in three structural proteins (C, M, and E) and seven nonstructural proteins (NS) that, in combination with cellular proteins, catalyze the synthesis of new viral RNA and the assembly of new viral particles. These virions are released by exocytosis to infect new naïve (uninfected) cells. Currently, only four small molecules have shown promising antiviral activity against DENV, including Chloroquine, Celgosivir, Balapiravir, and UV-4B9 that targets various steps of the viral replication cycle, as indicated in (**B**). Image of the *Orthoflavivirus* genome structure and viral polyprotein modified by ViralZone, SIB Swiss Institute of Bioinformatics (available at: https://viralzone.expasy.org/24 (accessed on 28 October 2025)).

This review article compiles and discusses natural compounds with reported antiviral activity against arboviruses, particularly flaviviruses such as all four dengue virus serotypes (DENV-1 to 4) and Zika virus (ZIKV). In addition, to complement the literature-based discussion, we performed molecular docking analyses of some natural compounds included as a series of illustrative images depicting these interactions. These in silico results are intended to exemplify and visualize the molecular interactions described in previous studies, not to present new experimental data. Flaviviruses are enveloped viruses with a positive-strand RNA genome of ~11 kb with one single open reading frame (ORF) encoding for three structural proteins—capsid (C), precursor membrane (prM), and envelope (E) proteins, and seven non-structural (NS) proteins NS1, NS2A, NS2B, NS3, NS4A, 2k peptide, NS4B and NS5 (Figure 1A). To infect host cells, the outer viral protein—*envelope*—interacts with its cognate receptor(s) at the cell surface, followed by virus endocytosis. Then, the fusion between the virion and the endosomal membranes leads to the release of the ribonucleocapsid into the cytoplasm, which results in uncoating the viral RNA, eventually translated into a single polyprotein by ribosomes on the endoplasmic reticulum (ER). The polyprotein is then cleaved by the viral protease NS3 and host proteases into structural and nonstructural proteins, all anchored to the ER membrane. Several nonstructural proteins, for instance, NS1, NS4A and NS4B assemble into the replication complex and drive the invagination of the ER membrane to produce replication organelles. In the replication complexes, the viral RNA-dependent-polymerase NS5 (RdRp) in combination with the cleaved NS2A and NS2B-NS3 complex catalyzes the synthesis of the viral RNA that is then packaged into new nucleocapsids and envelopes, creating newly immature virions. Immature virions enter the trans-Golgi Network (TGN) via their secretion in vesicles, where they progress through chambers of decreasing pH. Finally, to exit the host cell, a cellular protease called *furin* cleaves the precursor peptide (pr) of the pr-M protein, producing mature virions that will be released by exocytosis to infect new naïve (uninfected) cells (Figure 1B) [5]. The multifaced NS1 protein, the only secreted viral protein during flavivirus infection, facilitates new virus assembly in the ER and the TGN, and triggers immune evasion and pathogenic mechanisms after being secreted from infected cells [6,7].

Currently, no specific treatment exists to combat these arboviral infections. Treatment primarily focuses on symptom management, with no direct impact on viral replication. While few effective vaccines exist for a small number of arboviruses, several vaccine candidates are still under development, for instance, DENV and CHIK. For DENV only two vaccines have been licensed for their administration in humans living in endemic regions, and one more is under a current phase 3 trial [8]; however, certain safety pre-existing conditions must be fulfilled, such as the immune serostatus of those to be vaccinated before vaccination [9]. Despite all this evidence, no effective DENV vaccine is yet available [10,11].

With this unclear panorama, the development of small molecules with potential antiviral activity continues as a promising alternative to defeat these viral diseases; however, finding a suitable and effective drug against these important human arboviruses has been a slow process. To date, four small molecules with promising anti-DENV activity, including chloroquine (ClinicalTrials.gov Identifier: NCT00849602), celgosivir (NCT01619969, NCT02569827), balapiravir (NCT01096576), and UV-4B9 (NCT02061358, NCT02696291), have entered Phase I or Phase II clinical trials (Figure 1B). Despite these promising candidates, no effective antiviral drugs are currently available to treat the arboviral diseases impacting human public health systems worldwide. Today, a vast array of plant, animal, microbial, and marine species yields a plethora of natural products, with diverse chemical structures [12]. These compounds have been -and will remain- pivotal in drug discovery and development. Plant-derived systems, in particular, offer a rich source of lead compounds for healthcare applications. Recent advancements in elucidating the structures of plant-derived compounds have provided valuable insights into the development of novel drugs, including those targeting cancer, infections, and viral diseases; many natural products have shown particularly promising antiviral activity against arboviruses. Some important natural products and their possible interaction mechanism are discussed below [13]. Overall, this review presents the current state of knowledge of distinct groups of natural products with promising antiviral activity against arboviruses, particularly the flaviviruses DENV and ZIKV. It includes results from both in vitro and in vivo studies, as well as findings from human trials (Table 1). Furthermore, the structural diversity of these compounds, their viral targets, and their mechanisms of viral inhibition, supported by in silico analyses, are also discussed.

## 2. Drugs and Prodrugs with Antiviral Activity Against Arboviruses

Several existing drugs and prodrugs have demonstrated potential antiviral activity against arboviruses such as DENV, offering promising opportunities for drug repurposing [14]. Compounds such as chloroquine—originally developed for the treatment of malaria and certain inflammatory diseases; Balapiravir, a prodrug designed for hepatitis C virus (HCV) therapy, and Celgosivir and iminosugars like UV-4B, have shown inhibitory effects against DENV in vitro by targeting viral replication or viral protein maturation. These agents have also exhibited varying degrees of efficacy in vivo. A brief overview of some of these compounds, whether derived from natural sources or not, is presented below, followed by a more in-depth discussion of natural compounds that have recently demonstrated potential antiviral activity against arboviruses.

### 2.1. Chloroquine, Increasing the Endosomal pH to Hinder Virus Replication

**Figure 2:**
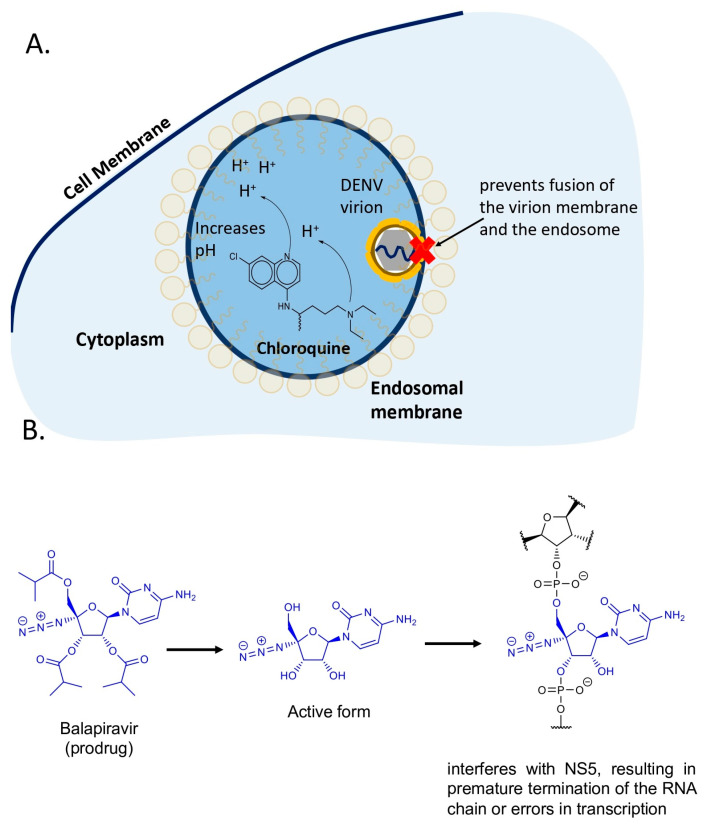
Molecular structure and mechanisms of Chloroquine and Balapiravir to inhibit flavivirus replication cycle. (**A**) Chloroquine is known to alkalinize the cellular endosomes by raising internal pH, interfering with the endosomal acidification, an essential step for viral genome release from viral capsid, affecting viral RNA replication and viral morphogenesis. (**B**) The active form of Balapiravir acts by inhibiting NS5, blocking viral replication, resulting in premature termination of the RNA chain or errors in transcription.

In 2013, a randomized, double-blind study evaluated the effects of chloroquine versus placebo over three days in 129 patients presenting with dengue-related symptoms. Among these, 37 were confirmed to have dengue and completed the study: 19 received chloroquine and 18 received placebo. The study found no significant differences in disease duration or fever intensity and duration. However, 63% of dengue patients treated with chloroquine (12 patients) reported a substantial reduction in pain intensity and a marked improvement in their ability to perform daily activities (*p* = 0.0004). These improvements were observed only during the medication period, with symptoms returning upon cessation. This effect was not noted in patients with other conditions. Consequently, the study suggests that chloroquine may enhance the quality of life for dengue patients by alleviating pain and facilitating daily activities. Nonetheless, as chloroquine did not impact overall disease duration or fever metrics, further research is necessary to confirm its clinical benefits and evaluate potential side effects in dengue patients [15]. A main limitation of the study is reflected in the reduced number of individuals examined. Chloroquine (Figure 2A) is known to affect intracellular exocytic pathways by increasing endosomal pH. By raising the pH of the endosome, chloroquine can interfere with the fusion process between the endosomal membrane and the viral envelope, preventing the release of the viral genetic material into the cytoplasm of the host cell [16].

### 2.2. Balapiravir, a Nucleoside Analog That Inhibits Viral RNA Polymerases

Balapiravir (Figure 2B), a prodrug of the nucleoside analog 4′-azidocytidine (R1479), was hypothesized to be effective against DENV due to its structural similarity to the hepatitis C virus (HCV) RdRp. In vitro studies demonstrated that balapiravir inhibited DENV replication across several serotypes and strains, with EC_50_ values ranging from 1.9 to 11 µM in human hepatoma (Huh-7) cells and from 1.3 to 6.0 µM in primary human macrophages and dendritic cells, as measured by RT-qPCR. Additionally, it exhibited significant antiviral activity in peripheral blood mononuclear cells (PBMCs) with EC_50_ values between 0.10 and 0.25 µM.

Despite achieving mean plasma exposures of 3.56 µM and 5.85 µM at dosages of 1500 and 3000 mg twice daily, respectively, the drug failed to significantly alter viral load, viremia kinetics, cytokine levels, or hematological and biochemical parameters in clinical trials. This ineffectiveness may be attributed to the timing of administration and cell-type specificity, with reduced efficacy in alveolar basal adenocarcinoma cells (A549), and hepatic-derived epithelial cells (Huh-7), as well as inactivity in a murine DENV infection model [17].

### 2.3. Celgosivir, Affecting Glucosidases at the Endoplasmic Reticulum

**Figure 3:**
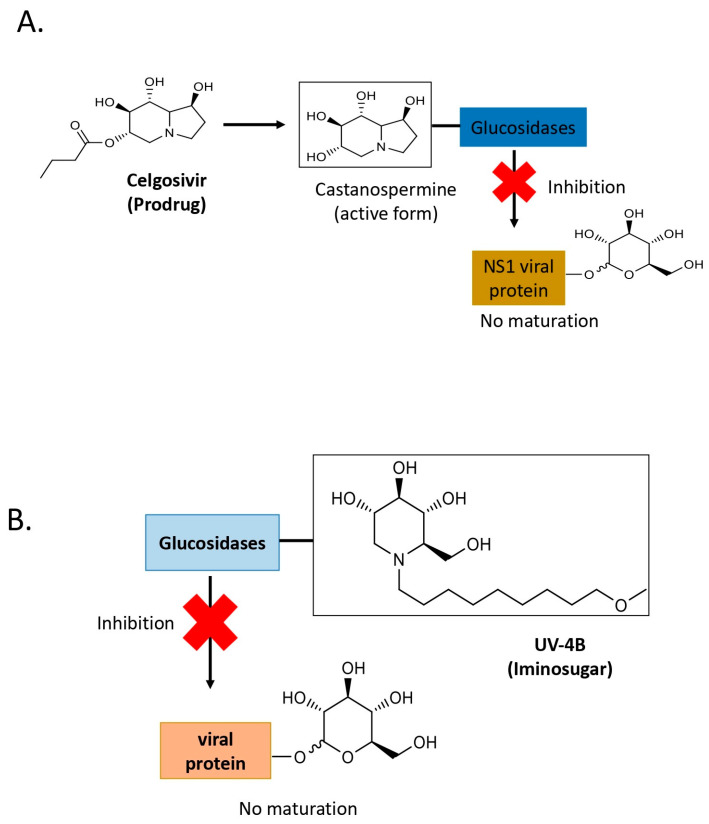
Molecular structure and mechanisms of Celgosivir and UV-4B to inhibit flavivirus replication cycle. (**A**) Castanospermine, the active form of Celgosivir, inhibits glucosidases that prevent NS1 maturation by inducing protein mis-glycosylation, leading to reduced secretion and altered protein biological activity. (**B**) UV-4B inhibits glucosidase I and II and interferes with the maturation of viral glycoproteins, including the envelope and the NS1 viral proteins.

In 2014, a study in vivo demonstrated a high protective efficacy of celgosivir (Figure 3A), a prodrug α-glucosidase I inhibitor, when administered twice daily to AG129 (type I interferon receptor knock-out) mice infected with DENV. Years later 2016, a similar regimen was tested in a clinical trial, which did not significantly reduce serum viral loads in patients [18]. This discrepancy likely stemmed from initiating treatment when patients were already viremic. To better replicate the clinical scenario, researchers employed patient-isolated viruses to develop new mouse models of DENV-1 and DENV-2 infections. These models were used to test the twice-daily treatment regimen, starting either on the day of infection or on the third day post-infection, when viremia peaked in the mice. Results indicated that treatment commenced on day 0 effectively reduced viral load, whereas no benefit was observed when treatment began on day 3. These findings suggest that in vivo antiviral efficacy diminishes once viremia reaches peak levels. Subsequently, a four-times-daily treatment regimen was tested and was found to significantly reduce viremia, implying potential efficacy in human clinical trials [19,20,21].

### 2.4. Iminosugars, Impacting Post-Translational Modifications to Inhibit Viral Proteins

Iminosugars such as UV-4B are host-targeted *glucomimetics*—chemical entities that mimic the structure or function of a native carbohydrate [22]—that inhibit endoplasmic reticulum α-glucosidase I and II enzymes, resulting in improper glycosylation and misfolding of viral glycoproteins. These compounds, UV-4B, have a broad-spectrum antiviral activity against diverse viruses, including DENV and influenza (Figure 3B) [23]. Regarding DENV, the antiviral efficacy of UV-4B was assessed against DENV-2 in human-relevant cell lines from liver (Huh-7), neurons (SK-N-MC), and skin (HFF-1). Following DENV infection and UV-4B treatment, infectious viral loads were quantified via plaque assays in Vero cells. Additionally, the effects on cell proliferation in both infected and uninfected cells were examined. UV-4B demonstrated antiviral activity across all tested cell lines, with EC_50_ values of 23.75 μM in Huh-7, 49.44 μM in SK-N-MC, and 37.38 μM in HFF-1. These findings indicate that UV-4B possesses substantial antiviral potential against DENV, effectively reducing viral replication in diverse physiologically relevant cellular scenarios [24]. Studies using purified enzymes, as well as in vitro and in vivo models, demonstrated that inhibition of ER α-glucosidases and not the glycosphingolipid pathway appears to be responsible for the antiviral activity of UV-4B against DENV. These studies support the clinical development of UV-4B currently in Phase 1 and planned for Phase 2 clinical trials [25].

## 3. Natural Compounds with Antiviral Activity Against Arboviruses

Natural compounds from plants, marine organisms, and microorganisms represent a valuable source of bioactive molecules with potential antiviral properties. Many of these metabolites, including flavonoids, terpenoids, alkaloids, and quinones, exhibit diverse mechanisms that interfere with viral entry, replication, or assembly. The study of these natural products not only provides insights into antiviral mechanisms but also offers promising scaffolds for drug development. This section summarizes key natural compounds reported to inhibit arboviruses and discusses their potential therapeutic relevance [26].

### 3.1. Quinones, a Group of Natural Compounds with Privileged Structures

**Figure 4:**
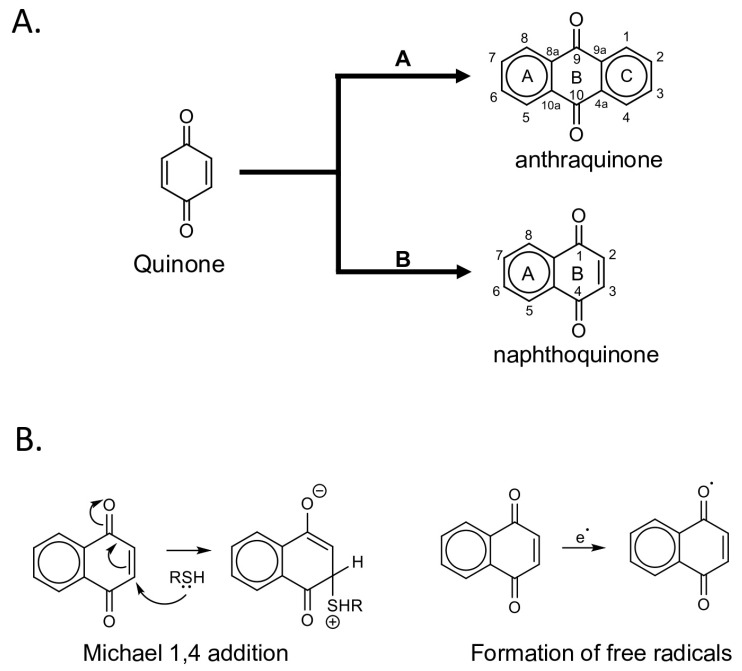
Molecular structures and antiviral mechanisms of Quinones. (**A**) Based on their molecular structure, *Quinones* are divided into *Anthraquinone* core, which contains a central anthracene nucleus, and *Naphthoquinone* core, which contains an aromatic ring attached to one of its sides. (**B**) *Napthoquinones* act via electrophilic Michael acceptors and redox-active interactions.

Quinones constitute an important group of natural compounds containing conjugated cyclic dione structure. In terms of their structure, they can be classified into *benzoquinones, naphthoquinones and anthraquinones* (Figure 4A). These structures play an important biological role due to their oxidation and reduction (redox) capacities, which can transport electrons in an organism through reversible oxidation-reduction (redox) reactions [27].

Quinones can exert several modes of action; for example, *naphthoquinones* are highly reactive in biological systems due to their ability to act as electrophilic Michael acceptors (1,4-addition), thereby neutralizing various nucleophilic bases present in cells or pathogenic agents. Additionally, their capacity to participate in 1,4-electrophilic addition reactions allows them to form covalent bonds (Figure 4B). This is particularly relevant because nitrogenous bases are susceptible to such reactions, which can inhibit viral genome replication or translation. Furthermore, the redox activity of quinones enables them to induce oxidative stress in virus-infected cells, leading to programmed cell death (apoptosis) in infected cells [28].

As for *anthraquinones*, in addition to their redox capacity, their functional groups around the aromatic ring enable π–π stacking and hydrophobic interactions with aromatic or nonpolar amino acid residues to inhibit viral protein and even the transcription and translation of the virus. Generally, this is the main mechanism of anthraquinones, through the incorporation of functional groups that allow generating strong electrostatic interactions (not necessarily covalent) in the amino acids or nitrogenous bases of the pathogen [29].

**Figure 5:**
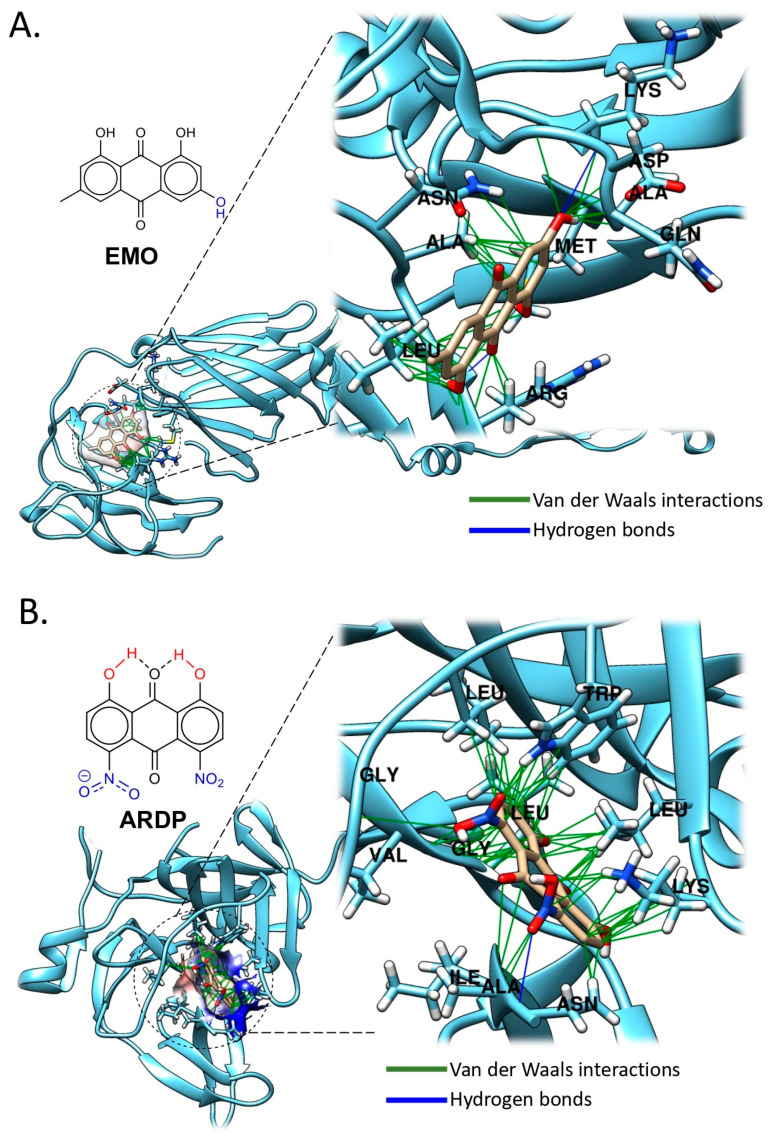
Molecular Docking Analysis of Natural Compounds with Viral protein targets. (**A**) Binding modes of EMO to the envelope protein domain (E-ZIKV) (PDB ID: 5JHM). The best binding pose corresponds to the lowest binding energy (Table S1). (**B**) Binding modes of ARDP006 to NS2B-NS3 complex (DENV2) (PDB ID: 2FOM) (Table S2). Polar residues (hydrogen bonds) are colored blue, and non-polar (Van der Waals, π–π) are green. Docking analysis was performed across the entire protein surface using AutoDock Vina (1.2.3-2). The best binding pose corresponds to the lowest binding energy (see Supplementary Table for validation analyses and docking methods).

*Emodin* (EMO), an anthraquinone derivative commonly found in natural plants, exhibits a broad spectrum of pharmacological effects, including antibacterial, anti-inflammatory, anti-fibrotic, anticancer, and antiviral properties. Chemically known as 1,3,8-trihydroxy-6-methylanthraquinone (C_15_H_10_O_5_), EMO (Figure 5A) is present in various plant families such as *Rheum palmatum*, *Polygonum cuspidatum*, *Polygonum multiflorum*, and *Cassiae semen*, among others. Recent evidence suggests that EMO displays promising antiviral activity and is frequently utilized in both treatment and prophylactic strategies against viral epidemics. EMO demonstrated substantial prophylactic efficacy against DENV-2 infection at two doses administered prior to infection. Additionally, it markedly reduced ZIKV infectivity by approximately 83.3% (from 7.8 × 10^3^ PFU/mL to 1.3 × 10^3^ PFU/mL) in Vero E6 cells [30].

It is hypothesized that in flaviviruses, EMO may similarly target regions of the viral membrane that exhibit transient exposure, local flexibility, or undergo membrane fusion events, thereby compromising virion stability or inhibiting viral entry [31] (Table 1).

To understand the structure–function relationship of EMO based on its reported inhibitory capacity, we performed a molecular docking analysis using the envelope protein of ZIKV (PDB ID: 5JHM) (Figure 5A). This analysis revealed that EMO binds within the domain III (DIII) of the ZIKV envelope protein, forming hydrophobic interactions with nonpolar amino acid residues such as Methionine-34 (Met34), Alanine-35 (Ala35) and -361 (Ala361), and Leucine-358 (Leu358), and -361 (Leu361). Moreover, notable interactions involving the carbonyl groups of EMO with Asn362 and Arg357 suggest the presence of polar contacts that may further stabilize the binding. These findings further support the hypothesis that EMO may act as an entry inhibitor by targeting the envelope protein and interfering with viral-host membrane fusion processes.

The marine environment has been extensively explored for novel pharmaceutical compounds, particularly antiviral agents. In this context, gymnochrome D (GYD), a polycyclic quinone, was isolated from *Gymnocrinus richeri* (Figure S1A), a crinoid species considered a living fossil. The antiviral efficacy of GYD against the DENV-1 virus (Hawaii/1944 strain) was assessed in vitro using a plaque reduction assay in porcine PS cells. Results demonstrated significant antiviral activity, with an RF_50_ value below 1 µg/mL (Table 1). Nevertheless, the precise mechanism underlying GYD’s antiviral effect remains undetermined, necessitating further mechanistic studies [32].

The DENV NS2B-NS3 protease is a critical drug target due to its role in viral replication within mammalian host cells. To date, over 20 inhibitors of this protease have been reported. However, their capacity to penetrate human cell membranes, where the viral protease is located, remains uncertain. Among the evaluated inhibitors, the *anthraquinone ARDP0006* (ARDP) exhibited the highest potency. Its CC_50_ values (K562: 63.5 µM, HuH-7: 106 µM), EC_50_ values (1.51 µM, 2.69 µM), and selectivity indices (42.1, 39.6) in both cell lines demonstrated its efficacy in inhibiting DENV replication at concentrations up to 10 µM without significant cytotoxicity (Table 1). Initially identified through virtual screening of a Mayo Clinic compound library, ARDP6 was further validated via biochemical DENV-2 NS2B-NS3 protease inhibition assays [33,34].

To complement the biochemical inhibition assays of ARDP against the NS2B-NS3 protease, we performed a molecular docking interaction (Figure 5B). Results showed that ARDP binds to chain B of the protease, interacting with polar amino acid residues such as Asparagine-152 (Asn152) and -167 (Asn167), and Lysine-74 (Lys74). These findings provide insight into how the nitro groups enhance the electrostatic affinity of the quinone, as illustrated by the surface Coulombic potential map. Additional relevant hydrophilic interactions involve residues Leucine-76 (Leu76), Tryptophan-83 (Trp83), Valine-147 (Val147), Glycine-148 (Gly148), and Isoleucine-165 (Ile165).

**Figure 6:**
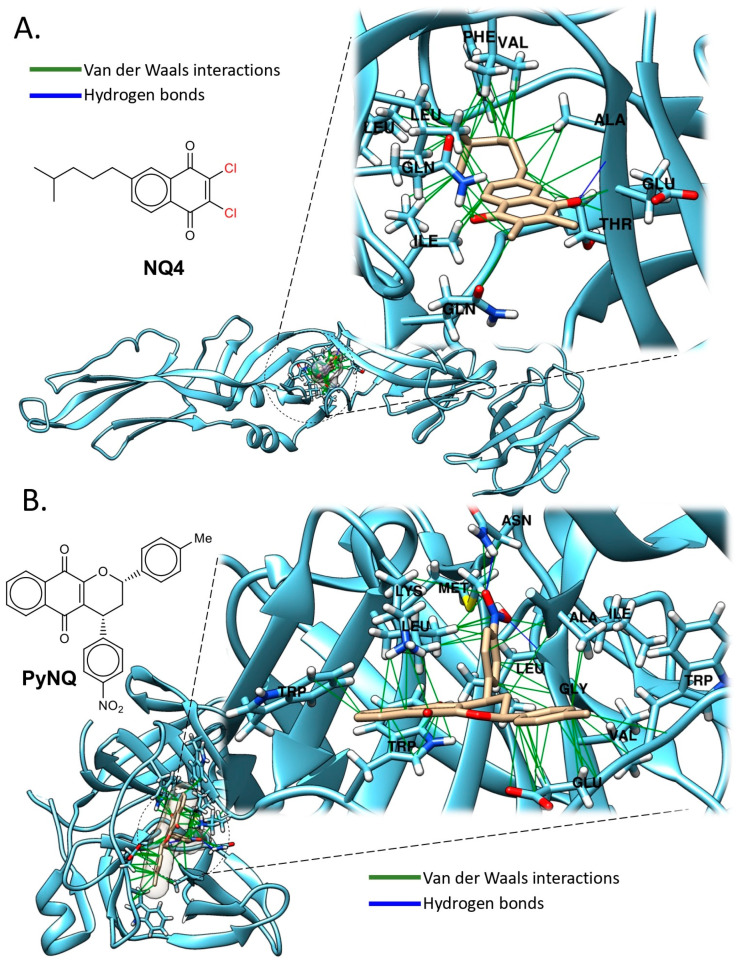
Molecular Docking Analysis of Natural Compounds with Viral protein targets. (**A**) Binding modes of NQ4 to the envelope protein domain (E-DENV2) (PDB ID: 1OKE) (Table S3). (**B**) Binding modes of PyNQ to NS2B-NS3 protease (E-DENV2) (PDB ID: 2FOM) (Table S4). Polar residues (hydrogen bonds) are colored blue, and non-polar (Van der Waals, π–π) are green. Docking analysis was performed across the entire protein surface using AutoDock Vina. The best binding pose corresponds to the lowest binding energy (see Supplementary Table for validation analyses and docking methods).

A series of synthesized terpenyl-1,4-naphthoquinones was evaluated in vitro against human herpesvirus types 1 (HHV-1) and 2 (HHV-2), as well as DENV-2. Using plaque-forming unit assays (PFU), cell viability tests, and molecular docking studies (this review), naphthoquinone NQ4 (Figure 6A) emerged as the most potent antiviral compound. NQ4 demonstrated significant antiviral activity against HHV-1 (EC_50_: <0.4 µg/mL, <1.28 µM) and DENV-2 (EC_50_: 1.6 µg/mL, 5.1 µM) at pre-infective stages (Table 1).

In silico analyses indicated that this quinone could bind to the prefusion form of the DENV-2 envelope glycoprotein (PDB ID: 1OKE). Docking analysis showed that the alkyl chain of NQ4 interacts with several nonpolar amino acid residues, including Alanine-50 (Ala50), Valine-130 (Val130), Leucine-135 (Leu135), -207 (Leu207), and -198 (Leu198), Phenylalanine-193 (Phe193), and Isoleucine-270 (Ile270). In contrast, the chloro (-Cl) groups form non-covalent interactions with the amide groups of two Glycine residues at -200 and -271 (Gln200, Gln271). These results highlight the importance of designing quinones with amphipathic properties. Overall, these findings emphasize the potential of NQ4 as an antiviral agent effective against both herpesvirus and dengue virus infections [35] (Table 1).

**Table 1:** Overview of natural products Targeting DENV and ZIKV Proteins: Docking Affinity and Mode of Action.

Compound	Source	EC_50_ or IC_50_	Active Against	Energy Affinity (Kcal/mol)	Mode of Action	Reference
EMO	Natural	3.2 µM	Envelope-ZIKV	−7.142	Inhibiting viral entry	[30]
GYD	Natural	0.8 µM	DENV1	ND	ND	[32]
ARDP	Derivative	1.5 µM	NS2B-NS3-DENV2	−8.167	Protease inhibition	[33]
NQ4	Derivative	5.1 µM	Envelope-DENV2	−7.496	Pre-infective stages	[35]
DTQ	Natural		DENV3	−43.6	NS5 MTase	[36]
PyNQ	Derivative	0.3 µM	NS2B-NS3	−9.404	Inhibit the ATPase activity	[37]
Bis-NQ1	Derivative	1.3 µM	ZIKV	ND	ND	[38]
Bis-NQ2	Derivative	0.6 µM	ZIKV	ND	ND	[38]
PSD	Derivative	1.3 µM	NS5-ZIKV	−27.4	RNA-dependent RNA polymerase	[39]
GBN	Natural	25 µM	DENV2	ND	ND	[40]
HPSD	Natural	21 µM	NS5 RdRp-DENV2	−7.425	Inhibits intracellular RNA synthesis	[41]
QCT	Natural	116 µM	NS5 RdRp-DENV2	−7.517	Inhibit cellular RNA polymerases	[42]
BCLN	Natural	23 µM	Envelope-DENV2	−8.645	Direct virucidal activity	[43]
BAC	Natural	10 µM	NS5 RdRp-DENV2	−8.596	Inhibits RNA synthesis	[44]
TERP-1	Natural	12 µM	NS5-DENV	ND	ND	[45]
TERP-2	Natural	3 µM	NS5-DENV	ND	ND	[45]
TERP-3	Natural	16 µM	NS5-DENV	ND	ND	[45]
ABF1	Derivative	10 µM	DENV2	ND	ND	[46]
ABF2	Derivative	1.4 µM	DENV2	ND	ND	[46]
MGT	Natural	1620 µM	NS2B-NS3-DENV2	−5.838	Protease inhibition and down-regulated NS1 expression	[47]
EMT	Natural	0.5 µM	DENV2	ND	targeting viral RNA synthesis or protein translation	[48]
PMT	Natural	26 µM	NS2B-NS3-DENV2	−7.443	Inhibited protease	[49]

A study on *Nigella sativa* quinones investigated DENV-3 NS5 methyltransferase (MTase) as a potential target for developing novel dengue therapies using in silico methods. Docking simulations with Discovery Studio software 3.1 indicated that dithymoquinone (DTQ) (Figure S1B) exhibited significant binding affinity (−43.6 Kcal/mol) to the active site of the target protein, comparable to the reference compound quercetin. Furthermore, ADMET analysis and Lipinski’s rule evaluation indicated that DTQ meets key pharmacokinetic and pharmacodynamic criteria. These results suggest that DTQ from *N. sativa* possesses anti-DENV activity, highlighting its potential for the development and optimization of dengue treatments [36].

Another study identified compounds with potential activity against DENV from a library of synthetic naphthoquinones. Briefly, various 1,4-pyranonaphthoquinones (PyNQ) were synthesized via a three-component reaction involving 2-hydroxynaphthoquinone, aldehydes (formaldehyde or arylaldehydes), and substituted dienophiles. These compounds were assessed for their ability to inhibit the ATPase activity of the viral NS3 enzyme in vitro and DENV replication in cultured cells. A 1,4-pyran naphthoquinone (1,4-PyNQ) (Figure 6B) was identified, demonstrating 99.0% inhibition of viral replication in mammalian cells, with an EC_50_ of 0.31 µM and significant reduction in NS3 ATPase activity [37] (Table 1).

Molecular docking analysis revealed that 1,4-PyNQ engages in hydrophobic and Van der Waals interactions with amino acid residues Methionine-49 (Met49), Triptophane-69 (Trp69) and -83 (Trp83), Leucine-76 (Leu76) and -149 (Leu149), and Valine-147 (Val147). Notably, Asn152 was the only residue found to form a hydrogen bond, specifically interacting with the NO_2_ group on the phenyl ring of the quinone. As previously observed for the anthraquinone ARDP, which also contains nitro groups, this finding suggests that the presence of NO_2_ moieties may enhance the electrostatic affinity of these compounds for the protease, potentially contributing to their inhibitory activity.

A study screened two series of bis-naphthoquinones for potential antiviral activity against Zika virus (ZIKV). A total of 27 compounds were evaluated in vitro using Vero cells. Among them, two compounds emerged as particularly promising. Compound 3,3′-((2-nitrophenyl)methylene)bis(2-hydroxynaphthalene-1,4-dione) (bis-NQ1) exhibited the highest selectivity index (SI = 1664), with a CC_50_ of 2297 µM and an IC_50_ of 1.38 µM. Meanwhile, 3,3′-(4-chlorophenylmethylene)bis(naphthalene-1,2,4-triyl triacetate) (bis-NQ2) showed notable antiviral activity with a CC_50_ of 483 µM, an IC_50_ of 0.65 µM, and an S.I. of 743 (Figure S1C,D) (Table 1). These results underscore the potential of bis-naphthoquinones as effective inhibitors of ZIKV replication, warranting further investigation into their mechanism of action and therapeutic development [38].

Recently, a study focused on the design, synthesis, and evaluation of anti-ZIKV activity of a series of anthraquinone analogs substituted with amine groups at the 7-position demonstrated moderate to excellent antiviral activity in most of the synthesized compounds. Notably, a compound phenylpiperidine-substituted derivative (PSD) exhibited EC_50_ values ranging from 1.33 µM to 5.72 µM and low cytotoxicity (CC_50_ > 50 µM) across various cellular models (Figure S1E) (Table 1). Moreover, this compound significantly improved survival rates in ZIKV-infected Ifnar1^−^/^−^ mice and reduced ZIKV-associated pathological damage. Molecular docking simulations indicated favorable interactions between the PSD compound and the RdRp NS5 of ZIKV, supporting its potential as a promising therapeutic candidate [39].

### 3.2. Flavonoids, Small Natural Compounds with Multiple Functions

Flavonoids constitute a broad class of plant-derived polyphenolic compounds, all sharing a characteristic benzo-pyrone backbone, and are primarily categorized into flavones, flavonols, and other subclasses based on structural variations. Widely distributed in fruits, vegetables, and medicinal plants, flavonoids play essential roles in plant defense and offer numerous health benefits in humans, including antioxidant, anti-inflammatory, and antiviral effects. Their relatively low molecular weight and structural diversity allow them to interact with a wide range of biological targets [50].

Constitute a substantial family of plant-derived polyphenolic compounds of low molecular weight, with a wide range of biological activities. In 2000, a study investigated the antiviral potential of various flavonoids extracted and characterized from the Mexican plants *Tephrosia madrensis*, *Tephrosia viridiflora*, and *Tephrosia crassifolia*, against DENV. The flavonoids glabranine (GBN) and 7-*O*-methyl-glabranine (7MGBN) demonstrated significant antiviral activity, achieving 70% inhibition of DENV at a concentration of 25 µM (Figure S2A,B) (Table 1). These findings suggest that both GBN and 7MGBN, isolated from *Tephrosia* species, possess a dose-dependent inhibitory effect on DENV in vitro, highlighting their potential as antiviral agents [40].

The aqueous extract of *Houttuynia cordata*, a common vegetable in Northern and Eastern Thailand, was evaluated for its antiviral activity against DENV-2. In vitro assays were conducted in three distinct modes: (i) protective, (ii) treatment, and direct blocking, utilizing HepG2 and LLC-MK2 cells. Plaque titration assays were used for modes (i) and (ii). In HepG2 cells, the extract significantly inhibited DENV-2 RNA production at concentrations of 10 μg/mL and 100 μg/mL. At the higher concentration, the extract effectively: (i) protected HepG2 cells from DENV-2 infection, (ii) revealed a decrease in infectious viral particles released to the culture medium.

In LLC-MK2 cells, the extract exhibited a protective effect on virion release within a 10–40 μg/mL concentration range (Table 1). High-performance liquid chromatography (HPLC) identified hyperoside (HPSD) as the major flavonoid component, likely responsible for the antiviral activity. The extract also prevented viral entry and inhibited viral processes post-adsorption. It is hypothesized that HPSD inhibits intracellular RNA synthesis by interacting with viral replication complex enzymes or proteins. Notably, isolated HPSD has not yet been tested as a pure compound [41].

**Figure 7:**
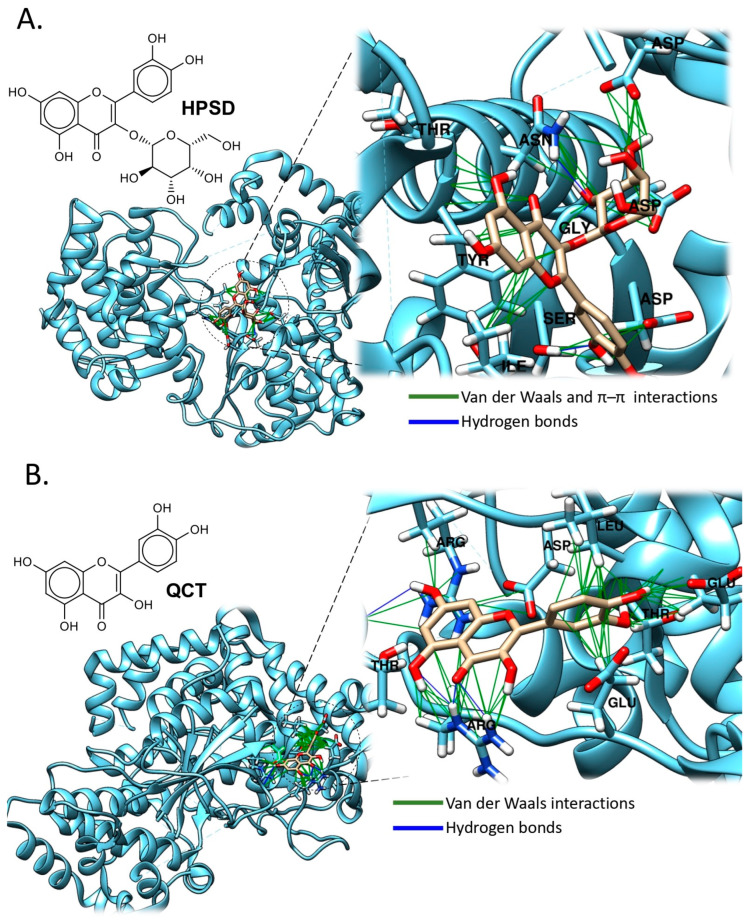
Molecular Docking Analysis of Natural Compounds with Viral protein targets. (**A**) Binding mode of HPSD to NS5 (DENV2) (PDB ID: 5K5M) (Table S5). (**B**) Binding mode of QCT to NS5 (DENV2) (PDB ID: 5K5M) (Table S6). Polar residues (hydrogen bonds) are colored blue, and non-polar (Van der Waals, π–π) are green. Docking analysis was performed across the entire protein surface using AutoDock Vina. The best binding pose corresponds to the lowest binding energy (see Supplementary Table for validation analyses and docking methods).

To elucidate the possible mechanism of action, we performed a molecular docking analysis (Figure 7A), which revealed that HPSD forms polar interactions with Aspartic Acid-664 (Asp664) and Histidine-798 (His798) through the hydroxyl groups of the phenyl substituent. Additionally, the hydroxyl groups on the sugar moiety establish hydrogen bonds with Serine-661 (Ser661), Aspartic acid-529 (Asp529), and -663 (Asp663). Regarding aromatic interactions, Tyrosine-607 (Tyr607) engages in π–π stacking with the central flavonoid core.

Zandi et al. evaluated the flavonoids Quercetin (QCT) (Figure 7B), naringenin, daidzein, and hesperetin against the DENV-2. The antiviral activity was assessed in vitro using Vero cells, with DENV replication quantified via Foci Reduction Neutralization Test (FRNT) and quantitative real-time polymerase chain reaction (qRT-PCR). Among these compounds, only quercetin demonstrated significant inhibitory activity, with an IC_50_ of 35.7 μg/mL (Table 1). The selective index for quercetin was 7.07 when infected cells were treated, and 8.74 when uninfected cells were treated continuously from 5 h before infection until 4 days post-infection. Although the exact antiviral mechanism of quercetin remains undetermined, it is hypothesized that its activity could be analogous to that of other flavonoids, which inhibit cellular RNA polymerases and interfere with RNA complex formation. Further investigation is required to elucidate the precise molecular interactions underlying quercetin’s antiviral effects against DENV-2 [42].

Molecular docking analysis of QCT revealed multiple hydrogen bonding interactions with the target protein (Figure 7B). A hydrogen bond is formed between the aromatic ring and the chiral hydrogen of Threonine-543 (Thr543), as well as with Glutamine-545 (Glu545). The adjacent hydroxyl groups near the flavonoid carbonyl interact with Argenine-362 (Arg362), while the hydroxyl group at position 8 (ring B) also forms a hydrogen bond with Argenine-595 (Arg595). Additionally, hydrophobic and Van der Waals interactions were observed between Glutamine-365 (Glu365) and Leucine-544 (Leu544) and the phenyl group of QCT. These findings underscore the capacity of flavonoids to engage in diverse interactions across multiple binding sites within the target protein, as demonstrated by compounds HPSD and QCT.

**Figure 8:**
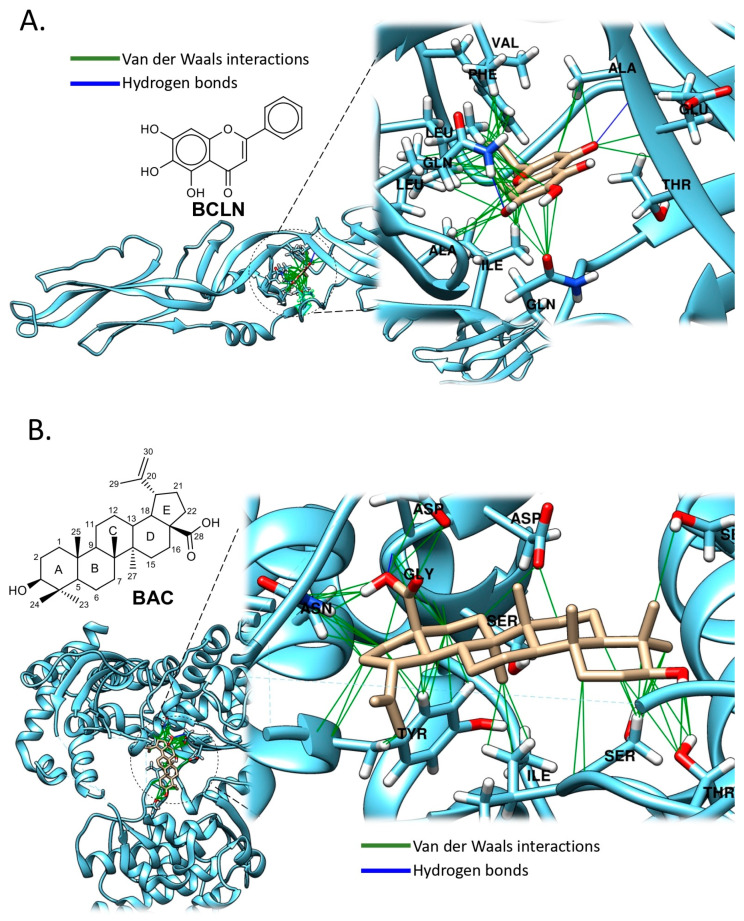
Binding Modes of Natural Compounds to DENV2 Targets. (**A**) Binding mode of BCLN to envelope protein (E-DENV2) (PDB ID: 1OKE) (Table S7). (**B**) Binding mode of BAC to NS5 RdRp (DENV2) (PDB ID: 5K5M) (Table S8). Polar residues (hydrogen bonds) are colored blue, and non-polar (Van der Waals, π–π) are green. Docking analysis was performed across the entire protein surface using AutoDock Vina. The best binding pose corresponds to the lowest binding energy (see Supplementary Table for validation analyses and docking methods).

*Scutellaria baicalensis*, a traditional Chinese medicinal herb of the *Lamiaceae* family, is notable for its root-derived flavonoid Baicalein (BCLN) (Figure 8A) [51]. An in vitro study utilizing Vero cells and the FRNT identified that BCLN inhibited DENV-2 replication in this cell line, exhibiting an IC_50_ of 6.46 µg/mL and a selectivity index of 17.8 when added post-adsorption. Additionally, the IC_50_ against DENV-2 was 5.39 µg/mL, with a selectivity index increasing to 21.3 when cells were treated pre-infection and continuously for 4 days post-infection. BCLN demonstrated direct virucidal activity with an IC_50_ of 1.55 µg/mL and anti-adsorption effects with an IC_50_ of 7.14 µg/mL (Table 1). These findings suggest that the extracellular and intracellular activities of BCLN against DENV-2 may involve its binding and/or inactivation of crucial structural and non-structural viral proteins, warranting further mechanistic studies [43].

Docking interaction analysis of compound BCLN with the DENV-2 envelope protein (Figure 8A) revealed that the compound binds within a hydrophobic pocket formed by residues Alanine-50 (Ala50), Valine-130 (Val130), Phenylalanine-193 (Phe193), Leucine-198 (Leu198), Alanine-205 (Ala205), Leucine-207 (Leu207), and Isoleucine-270 (Ile270). These hydrophobic and van der Waals interactions contribute to the stabilization of the flavonoid’s C-ring. Additionally, two polar interactions were identified between the hydroxyl groups on the A-ring and the residues Glycine-200 (Gln200) and -271 (Gln271). Notably, the docking occurred within the octyl β-D-glucopyranoside (BOG) pocket, despite the docking simulations being performed across the entire protein surface.

### 3.3. Terpenoids, a Group of Secondary Metabolites with Antiviral Properties

Terpenoids represent a diverse family of natural metabolites with well-documented biological activities, including antiviral properties. In this context, betulinic acid (BAC), a naturally occurring pentacyclic triterpenoid (Figure 8B), has demonstrated notable antiviral properties against DENV-2 in vitro. Initial cytotoxicity tests confirmed that BAC was non-toxic to Huh7, human liver cells at the concentrations tested, allowing for further antiviral evaluation. Dose-dependent inhibition studies revealed that treatment with 5 and 10 μM of BAC led to a significant reduction in DENV-2 viral titers, achieving approximately a 1.4 log_10_-fold decrease (Table 1).

Expanding the scope, the antiviral efficacy of BAC was assessed against other DENV serotypes (DENV-1, DENV-3, and DENV-4), as well as other positive-sense RNA viruses, including ZIKV and CHIKV. Similar inhibitory effects were observed across the three additional DENV serotypes at 5 and 10 μM concentrations, indicating broad-spectrum anti-dengue activity. Furthermore, treatment with BAC at 20 μM resulted in a 1.0 log_10_ and 1.3 log_10_ reduction in viral titers for ZIKV and CHIKV, respectively, highlighting its potential antiviral effects beyond flaviviruses. These findings suggest that BAC may possess a wider antiviral spectrum against RNA viruses, warranting further investigation into its mechanism of action and therapeutic potential [44].

The docking interaction analysis of BAC with the DENV-2 NS5 RdRp domain revealed that the compound docked with favorable binding affinity and interacted with residues located near the catalytic region of the enzyme. The compound formed polar interactions with residues Tyr607 and Asn610, as well as with Ser710, Thr794, and Ser796, both positioned near the priming loop region. Additionally, interactions were observed with negatively charged residues Asp663 and Asp664. Hydrophobic interactions were also noted, particularly with the nonpolar residue Ile797, located within the priming loop (Figure 8B). This suggests that BAC may exert its inhibitory effect by engaging multiple key residues across conserved motifs and the priming loop, potentially interfering with the initiation of viral RNA synthesis.

Additional terpenes have also been isolated from the bark and wood of *Trigonostemon cherrieri*, a rare plant from New Caledonia. Oxygenated terpenes, including compounds, here called TERP-1, -2 and -3 (Figure S3A–C), were evaluated for their ability to inhibit the purified DENV NS5 polymerase using an enzyme assay. The results demonstrated significant inhibitory effects, with IC_50_ values of 12.7 ± 0.2, 3.1 ± 0.2, and 16.0 ± 1.3 μM for compounds TERP-1, -2, and -3, respectively. However, the precise mechanisms underlying their inhibitory effects remain to be elucidated [45] (Table 1).

Finally, several natural and semisynthetic abietane-type diterpenoids have demonstrated significant antiviral activities (Figure S2C,D). A study reported the biological evaluation of various C-18- or C-19-functionalized known semisynthetic abietanes against ZIKV, DENV, Herpes simplex virus type 1, and CHIKV. Notably, the semisynthetic Abietane Ferruginol (ABF-1) and its analog 18-(phthalimid-2-yl) ferruginol (ABF-2) exhibited broad-spectrum antiviral properties. This compound displayed EC_50_ values ranging from 5.0 to 10.0 μM against Colombian Zika virus strains and an EC_50_ of 9.8 μM against CHIKV (Table 1). Given its activity against DENV-2 (EC_50_ = 1.4 μM, DENV-2), this ferruginol analog represents a promising broad-spectrum antiviral agent, paving the way for the development of novel antiviral therapies [46].

### 3.4. Phenolics, Aromatic Antioxidant Compounds with Potential Antiviral Activity

Phenolics are substances that possess an aromatic ring bearing one (phenol) or more (polyphenol) hydroxyl substituents, including functional derivatives (esters, methyl ethers, glycosides, etc.) [52]. Phenolic compounds have emerged as promising candidates in the search for novel antiviral agents against DENV [53]. A screening of 850 ethyl acetate extracts from Madagascan plants led to the isolation of several phenolic glycosides from *Flacourtia ramontchi*. The antiviral activity was assessed using enzyme assays with purified DENV NS5 polymerase. Compounds PGS-1 and PGS-2 (Figure S3D,E) exhibited the most significant activity among the evaluated phenolic derivatives, with moderate inhibition of DENV NS5 polymerase. The IC_50_ values were 9.3 ± 2.8 μmol/L for PGS-1 and 9.5 ± 5.0 μmol/L for PGS-2. Further studies are required to elucidate the precise mechanism of action of these compounds against DENV [54].

**Figure 9:**
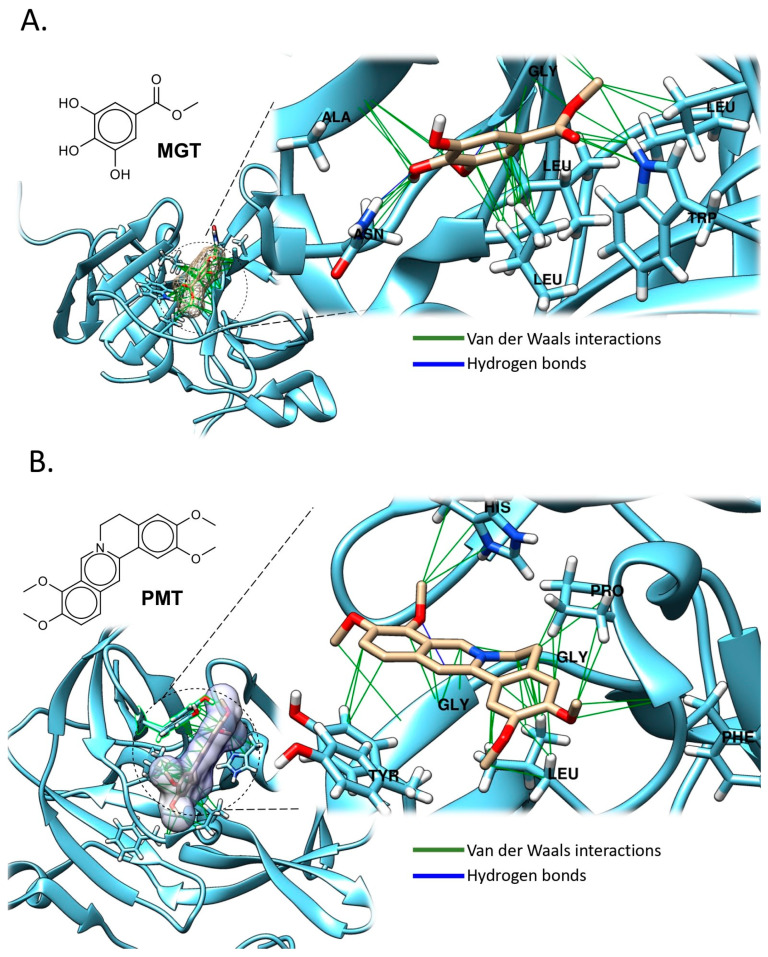
Binding Modes of Natural Compounds to DENV2 Targets. (**A**) Binding mode of MGT to NS2B-NS3 (DENV2) (PDB ID: 2FOM) (Table S9). (**B**) Binding mode of PMT to NS2B-NS3 complex (DENV2) (PDB ID: 2FOM) (Table S10). Polar residues (hydrogen bonds) are colored blue, and non-polar (Van der Waals, π–π) are green. Docking analysis was performed across the entire protein surface using AutoDock Vina. he best binding pose corresponds to the lowest binding energy (see Supplementary Table for validation analyses and docking methods).

A complementary study by Rahman and collaborators conducted an in vitro experiment using C6/36 cells via MTT colorimetric assay against DENV-2 and an enzyme assay with purified DENV-2 NS2B-NS3 protease. Methyl gallate (MGT), isolated from the methanol extract of *Quercus lusitanica*, inhibited 98% of DENV-2 NS2B-NS3 protease activity at 0.3 mg/mL (Table 1). Treatment of infected C6/36 cells with either crude methanol extracts or purified MGT down-regulated NS1 protein expression. The down-regulation may be responsible for the observed reduction or absence of cytopathic effects in treated infected cells, indicating significant antiviral activity (Figure 9A) [47].

To further understand the molecular basis of MGT’s inhibitory effect, docking interaction analyses were performed with the DENV NS2B-NS3 protease (Figure 9A). MGT was found to bind within a hydrophobic pocket formed by residues Leucine-76 (Leu76) and -85 (Leu85), Glycine-148 (Gly148), Alanine-64 (Ala64), and Asparagine-152 (Asn152). The binding was stabilized through multiple hydrophobic and van der Waals interactions, along with a key polar contact with Asn152, which may play a role in catalytic inhibition. These insights provide a foundation for the rational design of more potent phenolic-based inhibitors targeting viral proteases.

### 3.5. Alkaloids, Chemicals with Complex Chemical Structures as Promising Antiviral Candidates

Alkaloids represent a diverse group of naturally occurring compounds with significant potential as antiviral agents against arboviruses such as DENV. Their complex chemical structures enable interactions with multiple viral targets, making them promising candidates for drug development.

One such alkaloid, Emetine (EMT, Figure S3F), derived from *ipecacuanha*, has demonstrated potent antiviral activity against DENV at a low concentration of 0.5 μmol/L (277 ng/mL), as reported by Low and colleagues (Table 1). In vitro studies utilizing Huh-7 (human liver) and BHK21 (Hamster kidney) cell lines, combined with viral plaque assays, immunofluorescence, and RT-*q*PCR, confirmed that EMT dihydrochloride effectively inhibits DENV infection. This inhibition primarily occurs during the early stages of the viral replication cycle, likely targeting viral RNA synthesis or protein translation pathways. These results highlight emetine’s promising potential as an antiviral agent for dengue treatment [48].

In addition to EMT, *Coptis chinensis* Franch, a traditional medicinal plant widely used in China to treat bacterial, inflammatory, and fungal diseases, is known for its safety profile, exhibiting no significant side effects or toxicity at clinical doses. This plant is rich in Palmatine (PMT), which was evaluated for antiviral activity against DENV-2 in vitro using Vero cells through viral titer reduction assays. Palmatine demonstrated an EC_50_ of 26.4 μM and a selectivity index of 39, indicating promising antiviral potential. Additionally, PMT inhibited the NS2B-NS3 protease of WNV in enzyme assays (Table 1). However, its precise mechanism of action against DENV remains unclear, and further studies employing viral reverse genetics systems and investigations on virus-encoded proteases are underway to clarify its antiviral effects (Figure 9B) [49].

To further understand PMT’s mode of action, molecular docking analysis revealed that PMT binds with favorable affinity to the DENV NS2B-NS3 protease, interacting with key residues in both catalytic and allosteric binding sites (Figure 9B). The ligand formed pi-cation interactions with the catalytic residue Histidine-51 (His51), a critical component of the protease active site. Additionally, the compound engaged in hydrophobic interactions with nonpolar residues Leucine-128 (Leu128), Phenylalanine-130 (Phe130), and Proline-132 (Pro132), contributing to the stability of the docked complex. Polar interactions were also observed with Glycine-151 (Gly151), Gly153, and Triptophane-161 (Trp161)—residues located within the allosteric site—suggesting that PMT may exert inhibitory effects by binding to both the catalytic and regulatory regions of the enzyme. These multifaceted interactions highlight the potential of PMT as a non-competitive inhibitor targeting the NS2B-NS3 protease of DENV.

## 4. Conclusions

Arboviruses such as DENV, ZIKV, and CHIKV continue to spread, causing outbreaks worldwide each year. These viruses pose significant global health challenges due to the lack of effective treatments and the limited availability of vaccines. Natural compounds have emerged as promising candidates for antiviral therapy owing to their diverse biological activities and ability to interfere with viral replication and infection pathways.

In this review, we aimed to integrate the current knowledge on natural compounds with antiviral activity against clinically important human arboviruses, particularly DENV and ZIKV, with illustrative images depicting potential molecular interactions between these compounds and key viral proteins obtained through molecular docking analyses. The combination of biochemical assays and molecular docking studies provides visual and mechanistic insights into the possible binding modes and modes of action of these compounds.

Notably, molecular docking analyses revealed that compounds containing strong polar groups tend to interact with the viral NS2B/NS3 protease, while those with moderate polarity show stronger affinity for the envelope protein, and less polar compounds interact more effectively with the NS5 protein. The structural diversity and potent bioactivity of these natural compounds position them as promising leads for antiviral drug development and the design of new therapeutic molecules.

Overall, this review highlights the current state of antiviral compounds derived from natural sources against human arboviruses. Although several molecules have shown promising antiviral activity by reducing viral titers, no human studies have yet demonstrated effective treatments using these natural compounds against arbovirus transmission in endemic areas. Further clinical evaluations and investigations into combination therapies targeting both viral replication and the host immune response are still needed. Continued research integrating natural product chemistry, virology, and computational modeling holds strong promise for the development of effective antiviral strategies. Such multidisciplinary approaches not only enhance our understanding of antiviral potential but also have the capacity to accelerate the discovery of novel therapeutic agents to combat arboviral diseases.

## 5. Materials and Methods

This methods section describes the in silico analyses performed here to support the antiviral data reported for those antiviral compounds with inhibitory effect against some arboviruses such as DENV and ZIKV, and includes the different steps for molecular docking analyses. Of note, illustrative images included in Figure 5, Figure 6, Figure 7, Figure 8 and Figure 9 only correspond to those natural compounds with previously identified viral targets potentially involved in viral inhibition. Thus, docking analyses of only those molecules were performed to visualize these interactions.

### 5.1. Preparation of Proteins

Crystal structures of DENV and ZIKV proteins were acquired from the Protein Data Bank (PDB). PDB IDs: 5JHM (E-ZIKV), 2FOM (NS2B-NS3, DENV-2), 1OKE (Envelope DENV-2), 5K5M (NS5 RdRp, DENV-2) [38,54,55]. By removing all crystal water molecules, the protein structure was prepared for molecular docking processes. Small molecules were removed. The Protein and only the ligands were saved as two separate pdb files for validation docking.

### 5.2. Pocket Selection for Validation

The validation was carried out by re-docking the original ligand. The validation was performed in triplicate, and the best RMSD value (<2 Å) was selected for the docking of the molecules included in the review. Chimera 1.16 was used as visualization tool.

### 5.3. Ligand Selection and Preparation

The chemical structures of the small molecules were obtained from the PubChem databases. The 3D PDB structures were generated using Chimera software 1.16. The ligand molecules were subjected to energy minimization using Avogadro [56], a tool that applies the MMFF94 force field for energy minimization. Gasteiger charges were added to the small molecules using AutoDockTools 1.5.7.

### 5.4. Molecular Docking

AutoDock Vina [57] (Google Colab environment) was used to perform the molecular docking due to its accuracy and speed. AutoDockTools were used to prepare the input PDBQT files for the proteins and ligands. Polar hydrogens and Kollman charges were added to the three-dimensional structures. The grid box was set to cover the entire protein surface. The validation was performed at the active site of the crystallized ligand. In the case of the molecules used for docking, the analysis was conducted on the entire protein to determine whether the molecules exhibited better binding affinity at any other potential active site. It was confirmed that binding occurs at the known active site.

The following link provides access to the Google Colab environment where the docking simulations were carried out: https://colab.research.google.com/drive/1hHHQVtOpMfhp3a66Txdxp4IEbSVUADUl (accessed on 10 June 2025).

## Data Availability

All data is included in the submitted manuscript file.

## Supplementary Materials

The following supporting information can be downloaded at https://www.mdpi.com/article/10.3390/pathogens14111156/s1.

## Abbreviations

The following abbreviations are used in this manuscript:

DENV	Dengue virus
ZIKV	Zika virus
CHIKV	Chikungunya virus
NS	Non-structural proteins.
RdRp	RNA-dependent RNA polymerase
ER	Endoplasmic reticulum
TGN	Trans-Golgi Network
FRNT	Focus Reduction Neutralization Test
EMO	Emodin
GYD	Gymnochrome D
ARDP	Anthraquinone ARDP0006
DTQ	Dithymoquinone
PyNQ	1,4-pyranonaphthoquinones
GBN	Glabranine
7MGBN	7-O-methyl-glabranine
HPSD	Hyperoside
QCT	Quercetin
BCLN	Baicalein
MGT	Methyl gallate
BAC	Betulinic acid
EMT	Emetine
PMT	Palmatine
ABF	Ferruginol
TERP	Terpenes
