# Supplementary material for: Antiviral Compounds from Natural Sources Against Human Arboviruses: An Updated Review Including Illustrative In Silico Analysis
Source: Pathogens. 2025 Nov 13;14(11):1156. doi: 10.3390/pathogens14111156 (PMC12655658; doi:10.3390/pathogens14111156)
Supplement: Supplementary file 1 [file pathogens-14-01156-s001.zip › pathogens-3929123-supplementary.pdf]

Supplemental Material.

Antiviral Compounds from Natural Sources Against Human Arboviruses: An Updated Review Including illustrative *In Silico* analysis.

Julio Aguiar-Pech[1#], Henry Puerta-Guardo[2#], Rocío Borges-Argáez[1#]

[1]Unidad de Biotecnología, Centro de Investigación Científica de Yucatán, Calle 43 Número 130 x 32 y 34, CP 97205, Mérida, Yucatán, México

[2]Virology laboratory, Centro de Investigaciones Regionales "Dr. Hideyo Noguchi", Universidad Autónoma de Yucatán (UADY). 97225. Mérida, Yucatán, México.

[#]Corresponding authors: Julio Aguiar-Pech (aguiarjulio1@hotmail.com), Rocío Borges-Argáez (rborges@cicy.mx); Henry Puerta-Guardo (hpuertaguardo@gmail.com)

Table S1. Docking score of EMO vs E-ZIKV

Mode	Affinity (kcal/mol)	RMSD l.b. (Å)	RMSD u.b. (Å)
1	-7.142	0.000	0.000
2	-6.763	1.795	2.033
3	-6.479	1.204	3.319
4	-6.319	1.268	6.259
5	-6.232	38.200	40.480
6	-6.179	64.610	67.180
7	-6.122	38.320	41.910
8	-6.080	1.628	5.634
9	-4.250	37.430	40.040

Validation: Rigid receptor: E_ZIKV_Dockvalidation.pdbqt; Ligand: Emodin_.pdbqt; Grid center: X 5 Y -50 Z 15; Grid size: X 85 Y 40 Z 55; Grid space: 0.375; Exhaustiveness: 40; Performing docking (random seed: 711060146).

"Mode" refers to the ranking of the binding poses generated by the program according to predicted affinity.

"Affinity (kcal/mol)" corresponds to the predicted free binding energy of each pose — lower (more negative) values indicate stronger predicted binding.

"RMSD l.b. (Å)" (root mean square deviation lower bound) and "RMSD u.b. (Å)" (upper bound) indicate the deviation of each docking pose relative to the best-ranked pose, representing how similar or different the predicted conformations are; lower RMSD values (<2 Å) suggest consistent docking solutions.

Table S2. Docking score of ARDP vs NS2-NS3 DENV2

Mode	Affinity (kcal/mol)	RMSD l.b. (Å)	RMSD u.b. (Å)
1	-8.167	0.000	0.000
2	-7.981	1.938	4.616
3	-7.694	15.250	18.110
4	-7.619	14.670	18.320
5	-7.533	1.977	3.987
6	-7.475	15.090	17.750
7	-7.443	13.770	16.030
8	-7.387	15.650	18.380
9	-7.347	14.710	16.220

Validation: Rigid receptor: NS2B_NS3Dockvalidation.pdbqt; Ligand: ARDP006Q.pdbqt; Grid center: X -3 Y -15 Z 15; Grid size: X 45 Y 45 Z 45; Grid space: 0.375; Exhaustiveness: 40; Performing docking (random seed: -1652850905).

“Mode” refers to the ranking of the binding poses generated by the program according to predicted affinity.

“Affinity (kcal/mol)” corresponds to the predicted free binding energy of each pose — lower (more negative) values indicate stronger predicted binding.

“RMSD l.b. (Å)” (root mean square deviation lower bound) and “RMSD u.b. (Å)” (upper bound) indicate the deviation of each docking pose relative to the best-ranked pose, representing how similar or different the predicted conformations are; lower RMSD values (<2 Å) suggest consistent docking solutions.

Table S3. Docking score NQ4 vs E-DENV2

Mode	Affinity (kcal/mol)	RMSD l.b. (Å)	RMSD u.b. (Å)
1	-7.496	0.000	0.000
2	-7.465	4.289	6.244
3	-7.411	0.789	2.785
4	-7.262	1.970	3.932
5	-6.850	1.472	1.915
6	-6.789	1.939	2.296
7	-6.784	2.062	2.531
8	-6.682	3.660	5.343
9	-6.553	2.561	3.501

Validation: Rigid receptor: E_Dockvalidation.pdbqt; Ligand: NQ4_.pdbqt; Grid center: X -12 Y 75 Z 41; Grid size : X 40 Y 45 Z 111; Grid space : 0.375; Exhaustiveness: 40; Performing docking (random seed: 711060146).

“Mode” refers to the ranking of the binding poses generated by the program according to predicted affinity.

“Affinity (kcal/mol)” corresponds to the predicted free binding energy of each pose — lower (more negative) values indicate stronger predicted binding.

“RMSD l.b. (Å)” (root mean square deviation lower bound) and “RMSD u.b. (Å)” (upper bound) indicate the deviation of each docking pose relative to the best-ranked pose, representing how similar or different the predicted conformations are; lower RMSD values (<2 Å) suggest consistent docking solutions.

Table S4. Docking score PyNQ vs NS2-NS3 DENV2

Mode	Affinity (kcal/mol)	RMSD l.b. (Å)	RMSD u.b. (Å)
1	-9.404	0.000	0.000
2	-9.034	15.730	19.620
3	-9.011	9.454	12.440
4	-8.928	17.150	20.370
5	-8.823	17.900	21.760
6	-8.697	16.090	19.010
7	-8.602	15.370	18.990
8	-8.477	14.420	15.670
9	-8.455	14.050	17.870

Validation: Rigid receptor: NS2B_NS3Dockvalidation.pdbqt; Ligand: piranonaftoquinona.pdbqt; Grid center: X -3 Y -15 Z 15; Grid size: X 45 Y 45 Z 45; Grid space: 0.375; Exhaustiveness: 40; Performing docking (random seed: -1652850905).

"Mode" refers to the ranking of the binding poses generated by the program according to predicted affinity.

"Affinity (kcal/mol)" corresponds to the predicted free binding energy of each pose — lower (more negative) values indicate stronger predicted binding.

"RMSD l.b. (Å)" (root mean square deviation lower bound) and "RMSD u.b. (Å)" (upper bound) indicate the deviation of each docking pose relative to the best-ranked pose, representing how similar or different the predicted conformations are; lower RMSD values (<2 Å) suggest consistent docking solutions.

Table S5. Docking score HPSD vs NS5-DENV2

Mode	Affinity (kcal/mol)	RMSD l.b. (Å)	RMSD u.b. (Å)
1	-7.425	0.000	0.000
2	-7.373	22.370	25.280
3	-7.371	7.401	11.140
4	-7.202	2.690	4.287
5	-7.154	4.384	8.447
6	-7.107	2.002	3.281
7	-7.089	2.201	5.438
8	-7.025	14.830	18.830
9	-7.017	7.927	13.000

Validation: Rigid receptor: NS5Dockvalidation.pdbqt; Ligand: Hyperoside.pdbqt; Grid center: X -15 Y -39 Z -19; Grid size: X 60 Y 65 Z 60; Grid space: 0.375; Exhaustiveness: 40; Performing docking (random seed: -2086004631).

"Mode" refers to the ranking of the binding poses generated by the program according to predicted affinity.

"Affinity (kcal/mol)" corresponds to the predicted free binding energy of each pose — lower (more negative) values indicate stronger predicted binding.

"RMSD l.b. (Å)" (root mean square deviation lower bound) and "RMSD u.b. (Å)" (upper bound) indicate the deviation of each docking pose relative to the best-ranked pose, representing how similar or different the predicted conformations are; lower RMSD values (<2 Å) suggest consistent docking solutions.

Table S6. Docking score QCT vs NS5-DENV2

Mode	Affinity (kcal/mol)	RMSD l.b. (Å)	RMSD u.b. (Å)
1	-7.517	0.000	0.000
2	-7.420	34.260	36.280
3	-7.201	21.220	22.930
4	-7.176	1.926	7.052
5	-7.056	2.937	4.571
6	-7.052	56.600	58.680
7	-6.959	21.970	23.650
8	-6.931	15.350	17.800
9	-6.861	56.180	58.200

Validation: Rigid receptor: NS5Dockvalidation.pdbqt; Ligand: Quercetina.pdbqt; Grid center: X -15 Y -39 Z -19; Grid size: X 60 Y 65 Z 60; Grid space: 0.375; Exhaustiveness: 40; Performing docking (random seed: -2086004631).

"Mode" refers to the ranking of the binding poses generated by the program according to predicted affinity.

"Affinity (kcal/mol)" corresponds to the predicted free binding energy of each pose — lower (more negative) values indicate stronger predicted binding.

"RMSD l.b. (Å)" (root mean square deviation lower bound) and "RMSD u.b. (Å)" (upper bound) indicate the deviation of each docking pose relative to the best-ranked pose, representing how similar or different the predicted conformations are; lower RMSD values (<2 Å) suggest consistent docking solutions.

Table S7. Docking score BCLN vs E-DENV2

Mode	Affinity (kcal/mol)	RMSD l.b. (Å)	RMSD u.b. (Å)
1	-8.645	0.000	0.000
2	-7.807	3.170	5.428
3	-7.605	1.754	2.948
4	-7.481	1.547	3.196
5	-7.188	1.743	2.978
6	-7.069	41.260	42.780
7	-6.959	38.100	39.970
8	-6.952	31.810	32.550
9	-6.885	2.682	3.372

Validation: Rigid receptor: E_Dockvalidation.pdbqt; Ligand: Baicalein_.pdbqt; Grid center: X -12 Y 75 Z 41; Grid size : X 40 Y 45 Z 111; Grid space : 0.375; Exhaustiveness: 40; Performing docking (random seed: 711060146).

"Mode" refers to the ranking of the binding poses generated by the program according to predicted affinity.

"Affinity (kcal/mol)" corresponds to the predicted free binding energy of each pose — lower (more negative) values indicate stronger predicted binding.

"RMSD l.b. (Å)" (root mean square deviation lower bound) and "RMSD u.b. (Å)" (upper bound) indicate the deviation of each docking pose relative to the best-ranked pose, representing how similar or different the predicted conformations are; lower RMSD values (<2 Å) suggest consistent docking solutions.

Table S8. Docking score BAC vs NS5-DENV2

Mode	Affinity (kcal/mol)	RMSD l.b. (Å)	RMSD u.b. (Å)
1	-8.596	0.000	0.000
2	-7.969	20.310	25.610
3	-7.923	1.688	7.921
4	-7.839	16.130	20.270
5	-7.595	4.603	8.448
6	-7.524	11.280	15.250
7	-7.447	3.473	7.126
8	-7.431	13.740	17.950
9	-7.375	19.640	24.510

Validation: Rigid receptor: NS5Dockvalidation.pdbqt;Ligand: acido betulinico.pdbqt
Grid center: X -15 Y -39 Z -19; Grid size: X 60 Y 65 Z 60; Grid space: 0.375; Exhaustiveness: 40; Performing docking (random seed: -2086004631).

"Mode" refers to the ranking of the binding poses generated by the program according to predicted affinity.

"Affinity (kcal/mol)" corresponds to the predicted free binding energy of each pose — lower (more negative) values indicate stronger predicted binding.

"RMSD l.b. (Å)" (root mean square deviation lower bound) and "RMSD u.b. (Å)" (upper bound) indicate the deviation of each docking pose relative to the best-ranked pose, representing how similar or different the predicted conformations are; lower RMSD values (<2 Å) suggest consistent docking solutions.

Table S9. Docking score MGT vs NS2-NS3-DENV2

Mode	Affinity (kcal/mol)	RMSD l.b. (Å)	RMSD u.b. (Å)
1	-5.838	0.000	0.000
2	-5.804	1.748	5.089
3	-5.797	0.401	2.279
4	-5.640	1.834	4.737
5	-5.540	1.705	3.000
6	-5.443	1.604	2.542
7	-5.427	2.157	5.743
8	-5.291	2.107	5.159
9	-5.272	20.580	21.310

Validation: Rigid receptor: NS2B_NS3Dockvalidation.pdbqt; Ligand: Methyl gallate.pdbqt; Grid center: X -3 Y -15 Z 15; Grid size: X 45 Y 45 Z 45; Grid space: 0.375; Exhaustiveness: 40; Performing docking (random seed: -1652850905).

"Mode" refers to the ranking of the binding poses generated by the program according to predicted affinity.

"Affinity (kcal/mol)" corresponds to the predicted free binding energy of each pose — lower (more negative) values indicate stronger predicted binding.

"RMSD l.b. (Å)" (root mean square deviation lower bound) and "RMSD u.b. (Å)" (upper bound) indicate the deviation of each docking pose relative to the best-ranked pose, representing how similar or different the predicted conformations are; lower RMSD values (<2 Å) suggest consistent docking solutions.

Table S10. Docking score PMT vs NS2-NS3-DENV2

Mode	Affinity (kcal/mol)	RMSD l.b. (Å)	RMSD u.b. (Å)
1	-7.443	0.000	0.000
2	-7.382	6.980	11.320
3	-7.228	1.717	8.106
4	-7.194	1.512	8.430
5	-7.175	13.630	16.470
6	-7.166	1.636	3.666
7	-7.067	1.361	1.733
8	-7.001	1.394	7.749
9	-6.924	6.286	10.090

Validation: Rigid receptor: NS2B_NS3Dockvalidation.pdbqt; Ligand: Palmatine.pdbqt; Grid center: X -3 Y -15 Z 15; Grid size: X 45 Y 45 Z 45; Grid space: 0.375; Exhaustiveness: 40; Performing docking (random seed: -1652850905).

"Mode" refers to the ranking of the binding poses generated by the program according to predicted affinity.

"Affinity (kcal/mol)" corresponds to the predicted free binding energy of each pose — lower (more negative) values indicate stronger predicted binding.

"RMSD l.b. (Å)" (root mean square deviation lower bound) and "RMSD u.b. (Å)" (upper bound) indicate the deviation of each docking pose relative to the best-ranked pose, representing how similar or different the predicted conformations are; lower RMSD values (<2 Å) suggest consistent docking solutions.

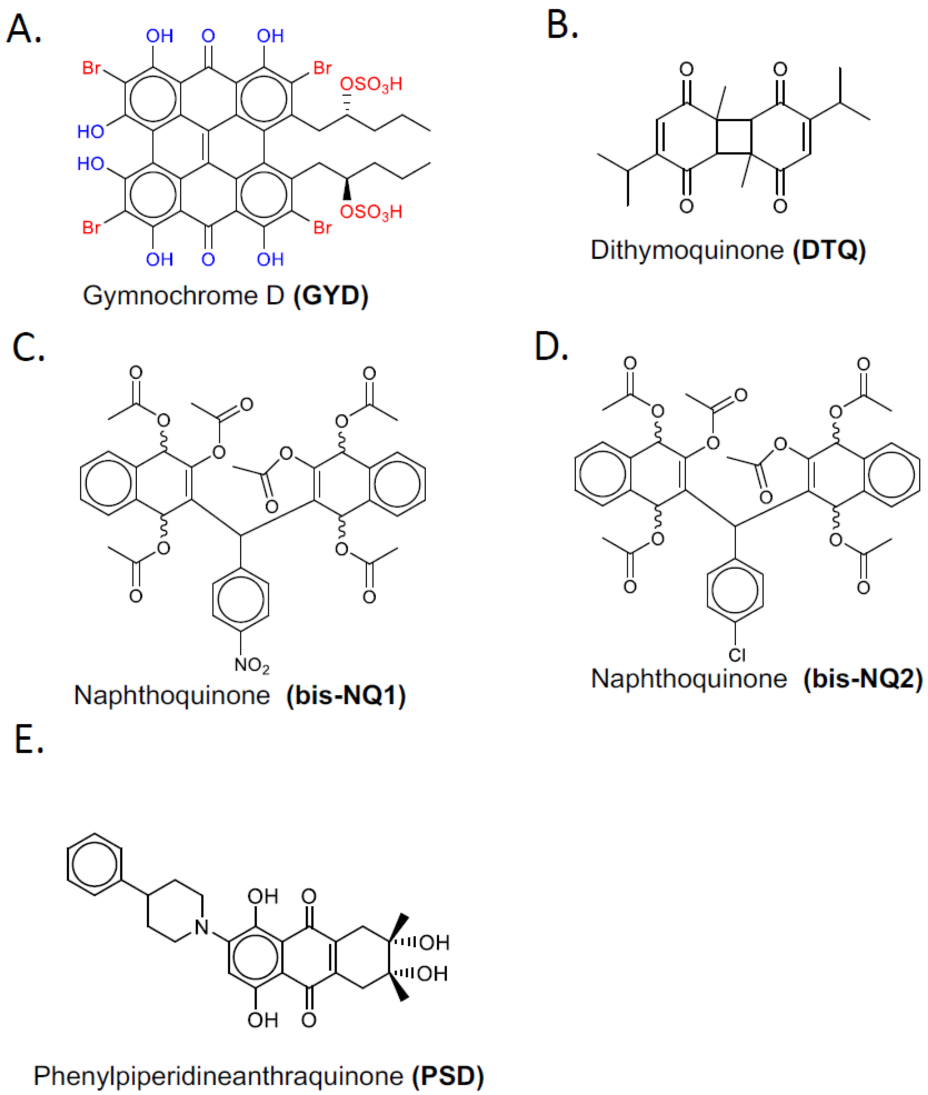

Figure S1. Antiviral Potential of Quinone Derivatives Against ZIKV. A) *Gymnochrome D* (GYD), a marine natural product isolated from the living fossil crinoids *Gymnocrinus richeri.* **B)** Dithymoquinone exhibited significant binding affinity to the active site of NS5. **C, D)** Inhibition of Zika Virus Replication by Synthetic Bis-Naphthoquinones with high selectivity index. **E)** a compound phenylpiperidine-substituted (PSD) derivative as a promising therapeutic candidate.

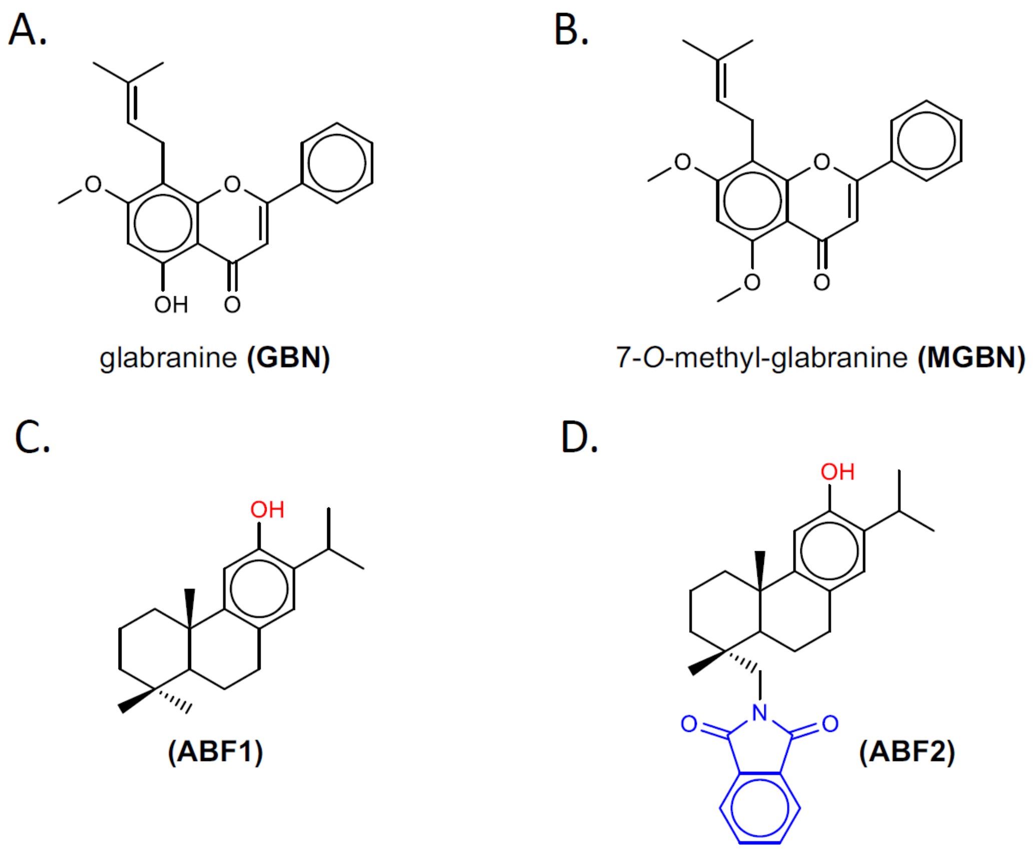

Figure S2. Structural Features and Antiviral Potential of Flavonoids Targeting DENV. A, B) GBN and MGBN effectively inhibited a viral replication of dengue virus offering a potentially acceptable alternative as a therapy for infections. **C, D)** Semisynthetic abietane ferruginol and its analogues (ABF1, 2) displayed broad-spectrum anti- viral properties against DENV, ZIKV and CHIKV.

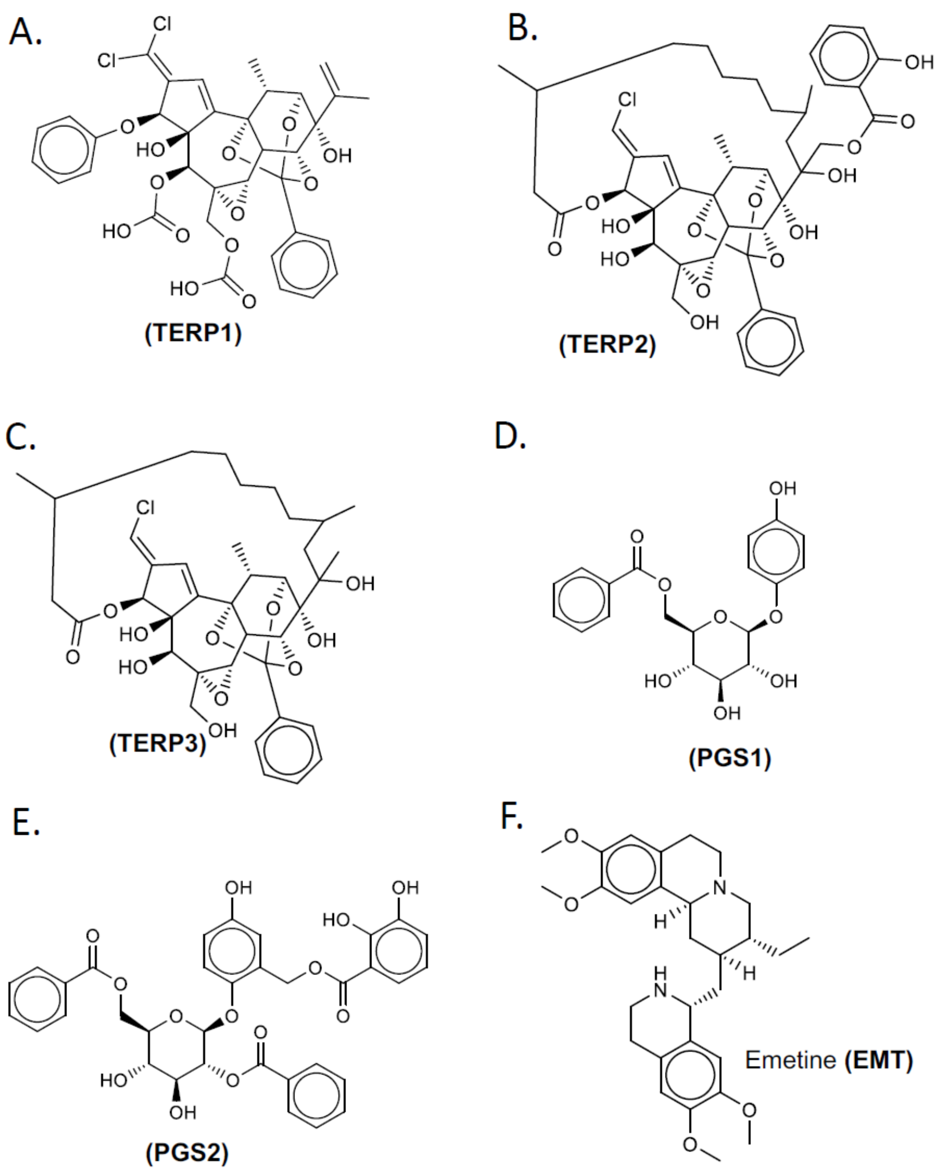

Figure S3. Antiviral Activity of terpenes Against DENV, ZIKV, and CHIKV. A-C) TERP-1, -2, and -3 are potent and selective inhibitors of NS5 DENV polymerase. **D, E)** Compounds PGS-1 and PGS-2 isolated from *Flacourtia ramontchi* showed modarate inhibition in the DENV RNA polymerase assay. **F)** EMT can inhibit DENV infection by impeding viral RNA synthesis therefore emetine could be further assessed and developed as a potential antiviral therapeutic agent.
